# Monitoring antimicrobial resistance in *Campylobacter* isolates of chickens and turkeys at the slaughter establishment level across the United States, 2013–2021

**DOI:** 10.1017/S0950268824000359

**Published:** 2024-02-26

**Authors:** Hamid R. Sodagari, Isha Agrawal, Mohammad N. Sohail, Setyo Yudhanto, Csaba Varga

**Affiliations:** 1 Department of Pathobiology, College of Veterinary Medicine, University of Illinois Urbana-Champaign, Urbana, IL, USA; 2 Carl R. Woese Institute for Genomic Biology, University of Illinois Urbana-Champaign, Urbana, IL, USA

**Keywords:** antimicrobial resistance, *Campylobacter coli*, *Campylobacter jejuni*, chickens, fluoroquinolone, macrolides, tetracycline, turkeys, United States

## Abstract

Foodborne infections with antimicrobial-resistant *Campylobacter* spp. remain an important public health concern. Publicly available data collected by the National Antimicrobial Resistance Monitoring System for Enteric Bacteria related to antimicrobial resistance (AMR) in *Campylobacter* spp. isolated from broiler chickens and turkeys at the slaughterhouse level across the United States between 2013 and 2021 were analysed. A total of 1,899 chicken-origin (1,031 *Campylobacter coli (C. coli)* and 868 *Campylobacter jejuni (C. jejuni)*) and 798 turkey-origin (673 *C. coli* and 123 *C. jejuni*) isolates were assessed. Chicken isolates exhibited high resistance to tetracycline (43.65%), moderate resistance to ciprofloxacin (19.5%), and low resistance to clindamycin (4.32%) and azithromycin (3.84%). Turkey isolates exhibited very high resistance to tetracycline (69%) and high resistance to ciprofloxacin (39%). The probability of resistance to all tested antimicrobials, except for tetracycline, significantly decreased during the latter part of the study period. Turkey-origin *Campylobacter* isolates had higher odds of resistance to all antimicrobials than isolates from chickens. Compared to *C. jejuni* isolates, *C. coli* isolates had higher odds of resistance to all antimicrobials, except for ciprofloxacin. The study findings emphasize the need for poultry-type-specific strategies to address differences in AMR among *Campylobacter* isolates.

## Introduction

*Campylobacter* is the most common enteric bacterial pathogen in humans in the United States of America (USA) causing an estimated 20 cases for every 100,000 persons each year [1] and is the leading cause of foodborne bacterial infections in the USA [2] and worldwide [3]. The majority of infections (estimated 90%) are caused by *Campylobacter jejuni (C. jejuni),* and only 5–10% are attributed to *Campylobacter coli (C. coli)* [1]. In addition to enteric diseases, *C. jejuni* has been linked to several post-infection complications such as irritable bowel syndrome, Guillain–Barré syndrome, and reactive arthritis [4]. Previous studies described the consumption of contaminated poultry products as a main source of campylobacteriosis [5, 6].

*Campylobacter* has become resistant to clinically important antimicrobials in human medicine and is therefore listed as a high-priority antimicrobial-resistant pathogen [7]. Recent studies from the USA [5], Switzerland [8], European Union (EU) [9], and South America [10] reported a high level of resistance in human *Campylobacter* isolates to tetracycline and ciprofloxacin. Macrolide resistance was reported worldwide; however, currently only a low level of resistance exists, which is an encouraging finding as macrolides (e.g. erythromycin) are the first drug of choice when treating campylobacteriosis.

Chickens and turkeys are important sources of antimicrobial-resistant *Campylobacter* [11, 12] as fluoroquinolone-and tetracycline-resistant *Campylobacter* isolates have been identified previously at chicken and turkey farms, slaughter plants, and retail stores in North America [11, 13–15] and worldwide [9, 16].

Antimicrobials have been used effectively for decades to treat, control, and prevent bacterial infectious diseases on poultry farms in North America [11, 13–15]; however, the use of antimicrobials has the highest impact on the emergence of antimicrobial resistance (AMR) [11, 14]. The US poultry sectors implemented antimicrobial use (AMU) reduction strategies and gradually eliminated the preventive use of medically important antimicrobials to contain the emergence of AMR. There are national [17], and global [18] initiatives to reduce the emergence and dissemination of antimicrobial-resistant commensal and pathogenic bacteria at the human–animal–environment interface. To limit the emergence of AMR in the USA, AMU as growth promoters in food-producing animals was prohibited, and the use of all clinically important antimicrobials in feed and water without the supervision of a licensed veterinarian was banned [19]. Similar bans have also been in effect in other regions, including Canada [11] and Denmark [20].

Evaluating AMR monitoring programmes to assess the current AMR patterns in foodborne pathogens to detect emerging AMR patterns and trends and assess the effectiveness of AMU policy changes over time is fundamental.

Considering all the issues presented above, publicly available data from the National Antimicrobial Resistance Monitoring System of Enteric Bacteria (NARMS) were evaluated to compare the prevalence of AMR in *C. jejuni* and *C. coli* isolated from the caecal content of chickens and turkeys at slaughter plants across the USA between 2013 and 2019. The provided information could assist public health and animal health authorities in developing effective antimicrobial stewardship programmes.

## Methods

### Study design

This study analysed publicly available AMR monitoring data collected by the NARMS, comprising data on AMR in *Campylobacter* spp. isolated from caecal samples obtained from chickens and turkeys at the United States Department of Agriculture (USDA) and Food Safety Inspection Service (FSIS)-regulated poultry slaughter establishments across the USA from 2013 to 2021.

Slaughter establishments were selected randomly by staff at FSIS considering their slaughter volume, type, and location. Once the number of samples per plant was established, caecal products were collected by pooling five samples of turkeys and chickens each into one sample [21].

### Laboratory testing

At the USDA FSIS Eastern Laboratory, standard microbiological methods were used to isolate *Campylobacter* strains from chicken and turkey samples [22]. Briefly, samples were enriched with buffered peptone water (BPW) and incubated at 42 ± 1.0°C for 29–31 h in a sealed, microaerobic environment. Subsequently, 30 μl from each well or test tube was streaked onto modified charcoal–cefoperazone–deoxycholate agar (mCCDA) plates and incubated at 42 ± 1.0°C for 22–24 h. Typical colonies from mCCDA were then streaked onto trypticase soy agar with 5% sheep blood agar (SBA) plates and incubated at 42 ± 1°C for 24–48 h. Confirmation of *Campylobacter* was done by examining the SBA plates and re-streaking if necessary for purity. The Bruker Matrix-Assisted Laser Desorption Ionization (MALDI) Biotyper was utilized to confirm the selected colonies. The latter were further tested for their antimicrobial susceptibility and speciated using whole-genome sequencing.

Antimicrobial susceptibility was determined using the Sensititre broth microdilution method using the CMV Campylobacter Selective Agar (CAMPY) plates. The following antimicrobials were tested: gentamicin, clindamycin, azithromycin, erythromycin, ciprofloxacin, nalidixic acid, and tetracycline. The interpretive guidelines for susceptibility testing and the categorization of resistant isolates were based on the minimum inhibitory concentration (MIC) values and breakpoints determined by the Clinical and Laboratory Standards Institute (CLSI) for *C. coli* and *C. jejuni* [23]. Supplementary Table 1 lists the breakpoints for both *C. coli* and *C. jejuni.* The AMR rate was categorized as rare (<0.1% of isolates), very low (0.1% to 1.0% of isolates), low (1.01%–10.0% of isolates), moderate (10.01%–20.0% of isolates), high (20.01%–50.0% of isolates), and very high (>50.0% of isolates) [24].

### Statistical analyses

STATA Intercooled software (Version 18, Stata Corporation, College Station, TX) and R software (Version 4.1.2 (2021-2111-01)) (R Core Team, 2020), within the RStudio platform (Version 1.4.1106 © 2009–2021 RStudio, PBC), were used for statistical analysis. The proportion of resistance to each antimicrobial was calculated by dividing the number of resistant isolates by the total number of isolates tested. For each proportion, the exact binomial 95% confidence intervals (CI) was calculated using the Clopper–Pearson methodology.

### Antimicrobial resistance pattern analysis

To analyse co-resistance and multidrug resistance patterns among antimicrobials and the clustering of resistant isolates, single-linkage dendrograms were created using Ward’s hierarchical clustering method, with Euclidean distances. Dendrograms were visualized in heatmaps using the heatmap.2 package in R software and the ggplot and RColorBrewer libraries.

To illustrate the pairwise and total correlations between AMRs, chord diagrams were created by using the chorddiag and devtools R-packages.

### Evaluating differences among campylobacter species, poultry type, and years

To determine differences in AMR between poultry species (chicken versus turkey), *Campylobacter* species (*C. coli* versus *C. jejuni*), and years (2013–2021), a multivariable logistic regression model for each antimicrobial was constructed. The dependent binary variable represented the resistance status (resistant = 1/susceptible = 0) of an antimicrobial, while the independent variables included poultry species (comparing turkeys to chickens), *Campylobacter* species (comparing *C. jejuni* to *C. coli*), and the study period (using the year 2013 to which all other years were compared). Statistically significant associations were signified by a *p*-value of ≤0.05 on the Wald *χ*2 test. For all model outcomes, odds ratios (ORs), 95% CIs, and *p*-values were presented. An OR less than 1 indicated a protective effect, while a value >1 signified that the variable had a positive effect on the dependent variable. For each model outcome, predicted probabilities were calculated and displayed graphically.

## Results

### Prevalence of antimicrobial resistance in *C. coli* and *C. jejuni* isolates of chickens and turkeys

A total of 1,899 *Campylobacter* isolates (1,031 *C. coli* and 868 *C. jejuni*) of chickens and 798 *Campylobacter* isolates (673 *C. coli* and 123 *C. jejuni*) of turkeys detected between 2013 and 2021 were included in this study.

In both *C. jejuni* and *C. coli* isolates derived from chicken caecal samples, there was a high prevalence of resistance to tetracycline (42–45%), moderate resistance to ciprofloxacin and nalidixic acid (17–22%), and low resistance to clindamycin and azithromycin (1–6%). On the other hand, in *C. coli* isolates, low resistance rates were observed for erythromycin and gentamicin (5–6%), while *C. jejuni* isolates showed very low resistance (0.2–1.0%) to the same antimicrobials (Table 1).

**Table 1.** Prevalence of antimicrobial resistance in *Campylobacter coli* (*n* = 1704) and *Campylobacter jejuni* (*n* = 991) isolates recovered from caecal samples of chickens and turkeys at the slaughterhouse level across the United States, 2013–2019

	Chicken			Turkey		
	C. coli	C. jejuni	Total	C. coli	C. jejuni	Total
	(*n* = 1,031)	(*n* = 868)	(*n* = 1899)	(*n* = 673)	(*n* = 123)	(*n* = 798)
Antimicrobial^a^	*N* (%)^b^ [CI]^c^	*N* (%) [CI]	*N* (%) [CI]	*N* (%) [CI]	*N* (%) [CI]	*N* (%) [CI]
AZI	59 (5.72)	14 (1.61)	73 (3.84)	82 (12.18)	3 (2.44)	85 (10.68)
	[4.38–7.32]	[0.882.69]	[3.02–4.81]	[9.81–14.90]	[0.50–6.96]	[8.62–13.03]
CIP	176 (17.07)	194 (22.35)	370 (19.48)	281 (41.75)	29 (23.58)	310 (38.94)
	[14.82–19.51]	[19.62–25.27]	[17.72–21.34]	[38.00–45.58]	[16.39–32.07]	[35.54–42.43]
CLI	66 (6.40)	16 (1.84)	82 (4.32)	98 (14.56)	10 (8.13)	108 (13.57)
	[4.98–8.07]	[1.06–2.98]	[3.45–5.33]	[11.98–17.45]	[3.97–14.44]	[11.26–16.14]
ERY	58 (5.62)	8 (0.92)	66 (3.47)	80 (11.89)	2 (1.63)	82 (10.30)
	[4.30–7.21]	[0.40–1.81]	[2.70–4.40]	[9.54–14.57]	[0.20–5.75]	[8.28–12.62]
GEN	53 (5.14)	2 (0.23)	55 (2.90)	126 (18.72)	5 (4.06)	131 (16.46)
	[3.87–6.67]	[0.03–0.83]	[2.19–3.75]	[15.84–21.88]	[1.33–9.23]	[13.95–19.22]
NAL	177 (17.17)	195 (22.46)	372 (19.59)	281 (41.75)	29 (23.58)	310 (38.94)
	[14.91–19.61]	[19.73–25.39]	[17.82–21.45]	[38.00–45.58]	[16.39–32.07]	[35.54–42.43]
TET	465 (45.10)	364 (41.93)	829 (43.65)	470 (69.84)	78 (63.41)	548 (68.84)
	[42.03–48.20]	[38.63–45.30]	[41.41–45.92]	[66.21–73.28]	[54.25–71.91]	[65.50–72.05]

a AZI, azithromycin; CIP, ciprofloxacin; CLI, clindamycin; ERY, erythromycin; GEN, gentamicin; NAL, nalidixic acid; TET, tetracycline.

b Number and percentage of isolates resistant to the antimicrobial.

c CI, exact binomial 95% confidence interval for the percentage of isolates resistant to the antimicrobial.

In both *C. jejuni* and *C. coli* strains isolated from turkey caecal samples, a very high resistance rate to tetracycline (63–69% of isolates) and a high resistance rate to ciprofloxacin and nalidixic acid (24–39%) were observed. Conversely, in *C. coli* isolates from turkeys, moderate resistance rates were observed for gentamicin, clindamycin, azithromycin, and erythromycin (12–19%), while *C. jejuni* isolates exhibited a low resistance rate (2–8%) to these antimicrobials (Table 1).

### Evaluating antimicrobial resistance patterns and clustering in *C. coli* and *C. jejuni* isolates of chickens

Hierarchical clustering dendrograms were constructed to evaluate the co-resistance patterns of the examined antimicrobials (columns) within bacterial isolates (rows).

**Figure 1.**
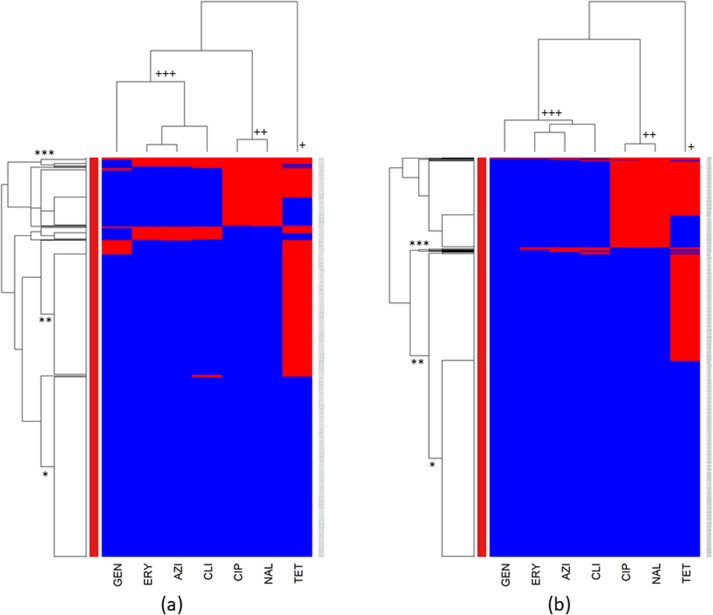
Clustering dendrogram (heatmap) of antimicrobial resistance in (a) *Campylobacter coli* and (b) *Campylobacter jejuni* isolated from chicken caecal samples at the slaughterhouse level across the United States, 2013–2021. AZI, azithromycin; CIP, ciprofloxacin; CLI, clindamycin; ERY, erythromycin; GEN, gentamicin; NAL, nalidixic acid; TET, tetracycline. Red colour: resistant; blue colour: susceptible.

The cluster analysis of AMR in *C. coli* and *C. jejuni* isolates from chickens is represented in Figure 1a,b, respectively.

In the columns of both heatmaps (Figure 1a,b), a primary cluster (marked with a plus sign (+)) was identified that signified resistance to tetracycline. The second cluster (++) indicated co-resistance to ciprofloxacin and nalidixic acid. The third cluster (+++) included co-resistance to gentamicin, erythromycin, azithromycin, and clindamycin. The rows of both heatmaps showed a cluster of isolates that were susceptible to all tested antimicrobials (*), another cluster of isolates that were susceptible to all antimicrobials except tetracycline (**), and a group (***) displaying resistance to most of the tested antimicrobials.

### Correlations among antimicrobial resistance

**Figure 2.**
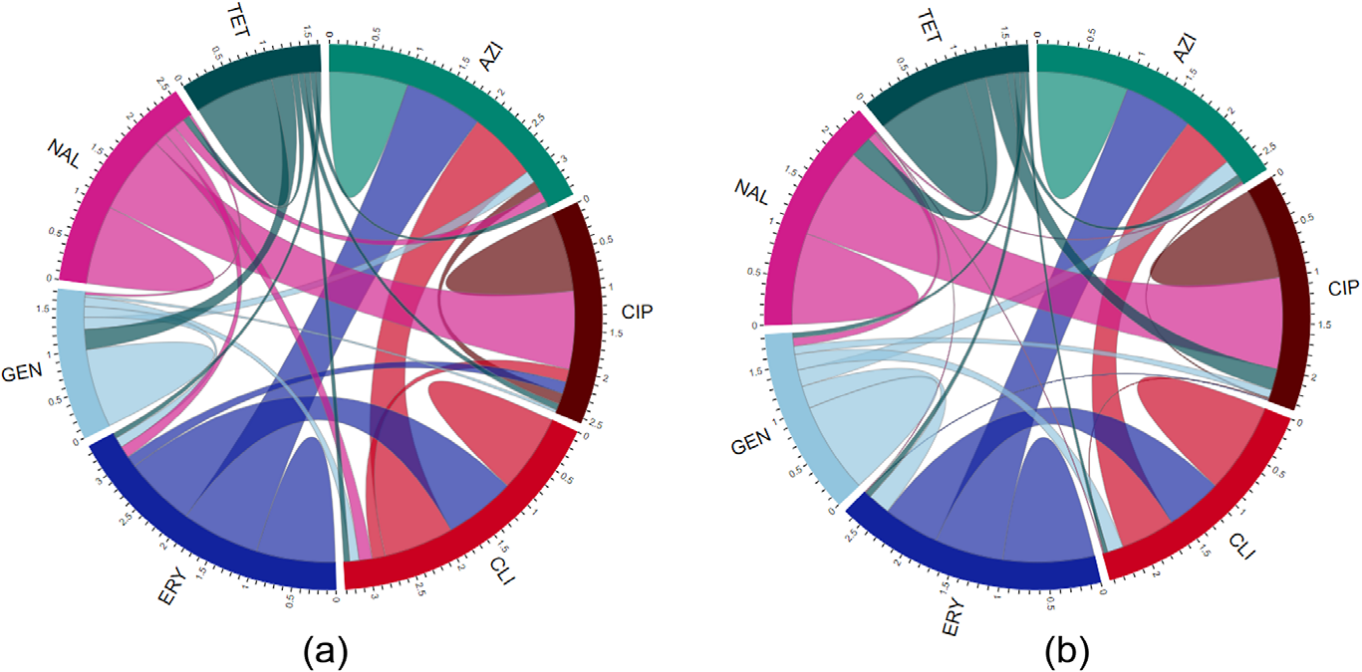
A chord diagram illustrating the pairwise and total correlations among antimicrobial resistance patterns in (a) *Campylobacter coli* and (b) *Campylobacter jejuni* isolated from chickens. AZI, azithromycin; CIP, ciprofloxacin; CLI, clindamycin; ERY, erythromycin; GEN, gentamicin; NAL, nalidixic acid; TET, tetracycline. Each antimicrobial agent is represented as a coloured segment, with the segment’s width reflecting the overall correlation of that antimicrobial agent. The network illustrates the connections and correlation strengths between different antimicrobial agents based on their resistance patterns. The thickness of the colour-coded chords in the diagram signifies the strength of the correlation between the resistance patterns of the respective antimicrobial agents.

The pairwise and total correlations among resistance to the examined antimicrobials in the *Campylobacter* isolates from chickens are illustrated in Figure 2. *C. coli* isolates from chickens (Figure 2a) exhibited strong total correlations for azithromycin (3.39) and erythromycin (3.38). The total correlations of other antimicrobials included clindamycin (3.31), ciprofloxacin (2.56), nalidixic acid (2.56), gentamicin (1.73), and tetracycline (1.63).

In *C. coli* isolates from chickens, strong positive pairwise correlations were found between ciprofloxacin and nalidixic acid (*ρ* = 0.99), azithromycin and erythromycin (*ρ* = 0.99), azithromycin and clindamycin (*ρ* = 0.89), and erythromycin and clindamycin (*ρ* = 0.89).

Among *C. jejuni* from chickens (Figure 2b), the highest total correlations were found for erythromycin (2.69) and azithromycin (2.65), similar to the *C. coli* isolates. The total correlations for other antimicrobials were clindamycin (2.44), nalidixic acid (2.36), ciprofloxacin (2.35), gentamicin (1.84), and tetracycline (1.71) (Figure 2b).

Strong positive pairwise correlations between ciprofloxacin and nalidixic acid (*ρ* = 0.99), azithromycin and erythromycin (*ρ* = 0.75), erythromycin and clindamycin (*ρ* = 0.61), and azithromycin and clindamycin (*ρ* = 0.59) were identified in *C. jejuni* isolates from chickens.

### Evaluating antimicrobial resistance patterns and clustering in *C. coli* and *C. jejuni* isolates of turkeys

The cluster analysis of AMR in *C. coli* and *C. jejuni* from turkeys is illustrated in Figure 2a,b, respectively.

Distinct clusters were observed in the columns of both heatmaps (Figure 2a,b). The first cluster, identified by the symbol (+), signified resistance only to tetracycline and the second cluster (++) indicated resistance to ciprofloxacin and nalidixic acid, while the third cluster (+++) included isolates that showed resistance to erythromycin, azithromycin, clindamycin, and gentamicin.

**Figure 3.**
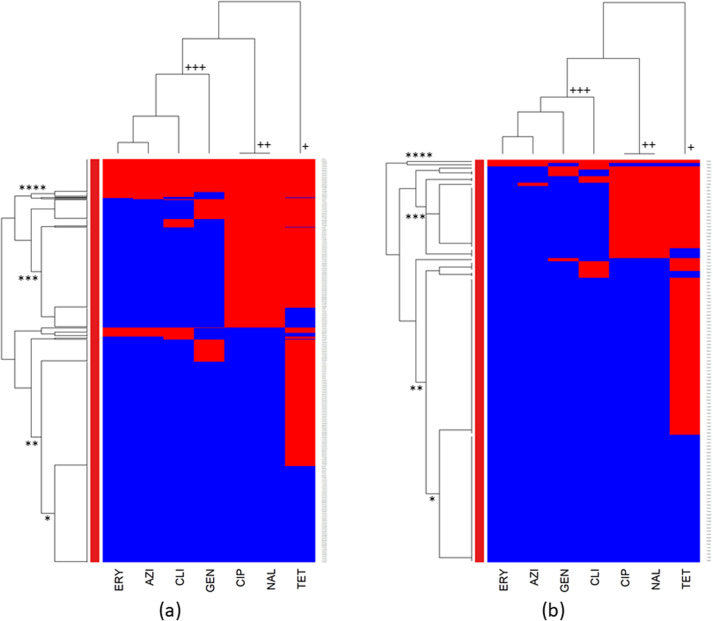
Clustering dendrogram (heatmap) of antimicrobial resistance in (a) *Campylobacter coli* and (b) *Campylobacter jejuni* isolated from turkey caecal samples at the slaughterhouse level across the United States, 2013–2021. AZI, azithromycin; CIP, ciprofloxacin; CLI, clindamycin; ERY, erythromycin; GEN, gentamicin; NAL, nalidixic acid; TET, tetracycline. Red colour: resistant; blue colour: susceptible.

Both *Campylobacter* species isolates illustrated in the rows of the heatmaps (Figure 3a,b) exhibited identical clustering patterns. The primary cluster (*) contained isolates that showed susceptibility to all tested antimicrobials, and the second cluster (**) contained isolates susceptible to all antimicrobials tested except for tetracycline. The third cluster (***) comprised isolates resistant to ciprofloxacin, nalidixic acid, and tetracycline, while the fourth cluster (****) comprised isolates resistant to all tested antimicrobials, thereby signifying a multidrug-resistant (MDR) group.

### Correlations among antimicrobial resistance

**Figure 4.**
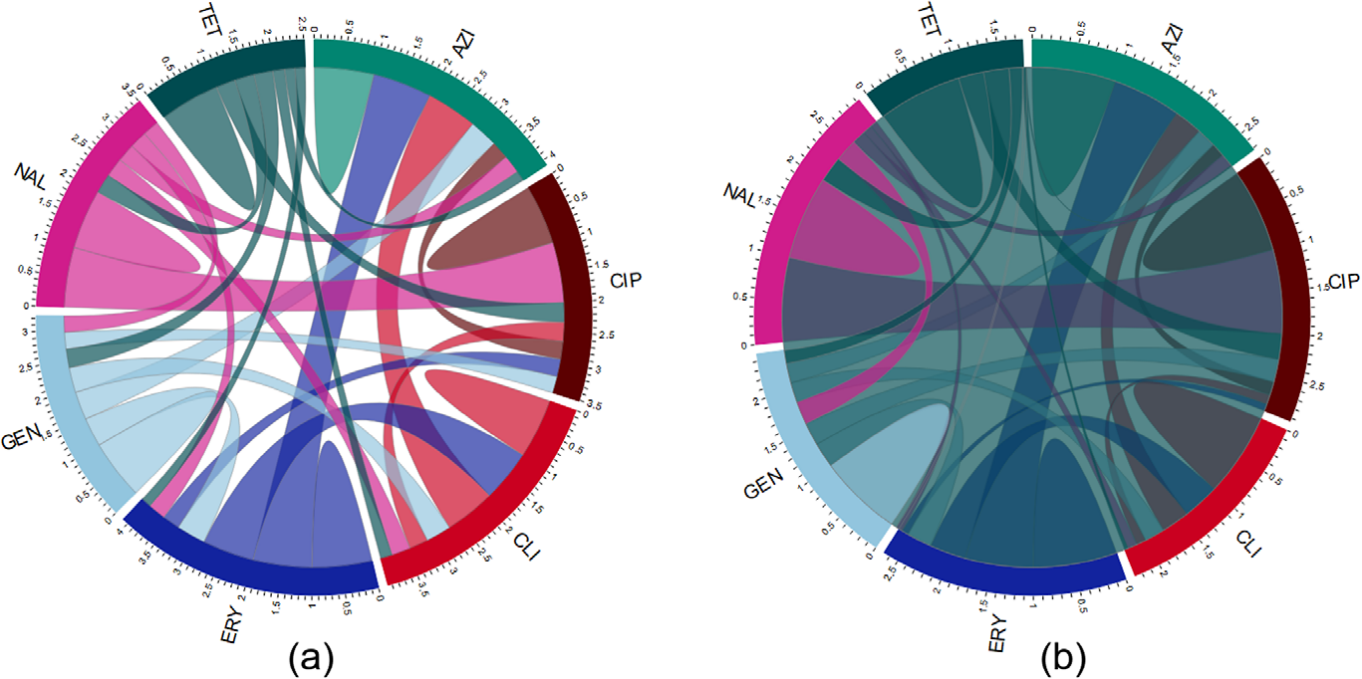
A chord diagram illustrating the pairwise and total correlations among antimicrobial resistance patterns in (a) *Campylobacter coli* and (b) *Campylobacter jejuni* isolated from turkeys. AZI, azithromycin; CIP, ciprofloxacin; CLI, clindamycin; ERY, erythromycin; GEN, gentamicin; NAL, nalidixic acid; TET, tetracycline. Each antimicrobial agent is depicted as a coloured segment, with the segment’s width reflecting the overall correlation of that antimicrobial agent. The network illustrates the connections and correlation strengths between different antimicrobial agents based on their resistance patterns. The thickness of the colour-coded chords signifies the strength of the correlation between the resistance patterns of the respective antimicrobial agents. The blurring effect observed in (b) is attributed to the negative correlation between erythromycin and tetracycline.

The pairwise and total correlations among resistance to the examined antimicrobials in the *Campylobacter* isolates from turkeys are illustrated in Figure 4.

In *C. coli* isolates (Figure 4a), the highest total correlations were detected for azithromycin (4.09) and erythromycin (4.09). Other antimicrobials showed the following total correlations: clindamycin (3.96), ciprofloxacin (3.51), nalidixic acid (3.51), gentamicin (3.24), and tetracycline (2.51) (Figure 4a). Strong pairwise correlations were detected between ciprofloxacin and nalidixic acid (*ρ* = 1), azithromycin and erythromycin (*ρ* = 0.99), azithromycin and clindamycin (*ρ* = 0.85), and erythromycin and clindamycin (*ρ* = 0.86).

In *C. jejuni* isolates (Figure 4b), ciprofloxacin (2.93) and nalidixic acid (2.93) showed the highest total correlations. Other antimicrobials had the following total correlations: azithromycin (2.71), erythromycin (2.67), gentamicin (2.48), clindamycin (2.28), and tetracycline (1.78) (Figure 4b). All pairwise correlations were positive, except erythromycin and tetracycline (*ρ* = −0.04). Strong positive pairwise correlations were identified between ciprofloxacin and nalidixic acid (*ρ* = 1), and azithromycin and erythromycin (*ρ* = 0.81).

### Evaluating differences among antimicrobial resistance in *Campylobacter* species, poultry types, and years

The probability of resistance to all tested antimicrobials (except for tetracycline) was significantly reduced during the study period when compared to 2013 (Table 2). The predicted probabilities of AMR across the study period considering poultry species and *Campylobacter* species are illustrated in Figure 3. Prediction for nalidixic acid was not illustrated as it was identical to ciprofloxacin.

**Table 2.** Multivariable logistic regression models representing the probability of resistance to antimicrobials among two *Campylobacter* species (*n* = 2,695) isolated from chicken and turkey samples at the slaughterhouse level across the United States, 2013 to 2021

	Antimicrobial^c^																	
	AZI			CIP			CLI			ERY			GEN			NAL		
Year	Odds ratio	95% CI^b^	*P*-value^c^	Odds ratio	95% CI	*P*-value	Odds ratio.	95% CI	*P*-value	Odds ratio	95% CI	*P*-value	Odds ratio	95% CI	*P*-value	Odds ratio	95% CI	*P*-value
2013	Referent	–	–	–	–	–	–	–	–	–	–	–	–	–	–	–	–	–
2014	0.72	0.27–1.97	0.529	0.56	0.28–1.13	0.107	0.73	0.27–1.99	0.542	0.72	0.26–1.97	0.527	0.70	0.27–1.78	0.455	0.60	0.30–1.20	0.151
2015	1.02	0.37–2.80	0.967	0.43	0.20–0.96	0.039	1.04	0.38–2.86	0.941	0.87	0.31–2.48	0.797	0.68	0.24–1.90	0.458	0.43	0.20–0.96	0.039
2016	0.41	0.12–1.42	0.163	0.46	0.22–0.97	0.041	0.31	0.08–1.18	0.086	0.42	0.12–1.44	0.168	0.30	0.90–1.02	0.053	0.46	0.22–0.97	0.041
2017	0.60	0.26–1.34	0.215	0.43	0.25–0.75	0.003	0.62	0.28–1.40	0.251	0.57	0.25–1.31	0.187	0.25	0.11–0.58	0.001	0.44	0.25–0.77	0.004
2018	0.47	0.22–1.03	0.059	0.54	0.32–0.90	0.019	0.44	0.20–0.95	0.038	0.41	0.18–0.90	0.026	0.28	0.13–0.59	0.001	0.55	0.33–0.91	0.021
2019	0.39	0.18–0.84	0.016	0.60	0.36–0.99	0.045	0.49	0.23–1.04	0.064	0.41	0.19–0.88	0.023	0.41	0.20–0.83	0.013	0.60	0.36–0.99	0.044
2020	0.44	0.20–1.00	0.050	0.62	0.37–1.06	0.080	0.60	0.28–1.32	0.207	0.44	0.19–1.00	0.049	0.41	0.20–0.88	0.021	0.62	0.37–1.06	0.080
2021	0.29	0.12–0.68	0.004	0.47	0.27–0.80	0.006	0.53	0.24–1.17	0.118	0.25	0.10–0.62	0.003	0.27	0.12–0.60	0.001	0.46	0.27–0.79	0.005
Poultry type^d^	2.53	1.78–3.58	<0.001	2.65	2.18–3.23	<0.001	2.85	2.06–3.93	<0.001	2.54	1.77–3.64	<0.001	4.88	3.44–6.92	<0.001	2.65	2.17–3.23	<0.001
*Campylobacter* species^e^	0.28	0.17–0.48	<0.001	1.05	0.86–1.29	0.628	0.37	0.24–0.57	<0.001	0.17	0.09–0.33	<0.001	0.10	0.05–0.22	<0.001	1.06	0.86–1.29	0.590

a AZI, azithromycin; CIP, ciprofloxacin; CLI, clindamycin; ERY, erythromycin; GEN, gentamicin; NAL, nalidixic acid.

b CI, exact binomial 95% confidence interval.

c Statistically significant at *P* ≤ 0.05.

d Turkeys versus chicken.

e *C. jejuni* versus *C. coli.*

**Figure 5.**
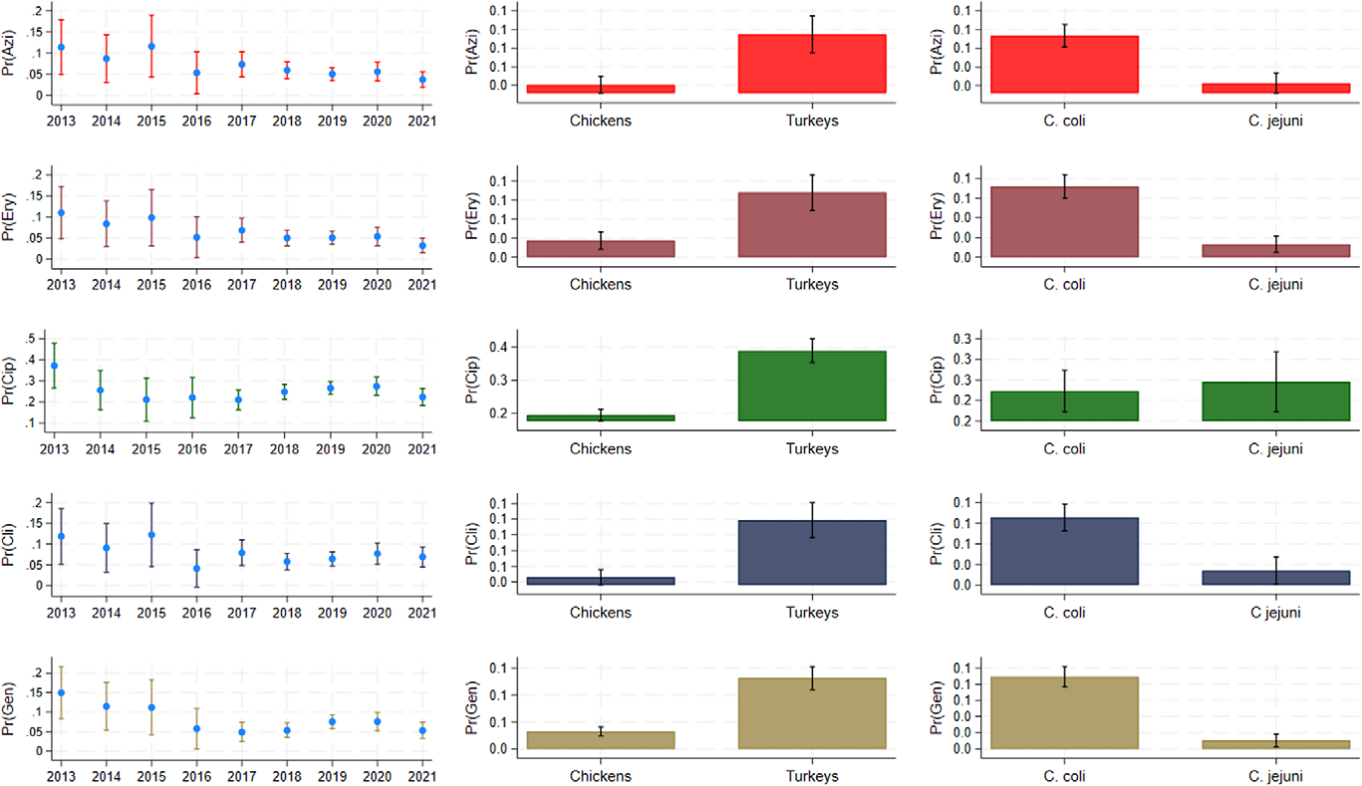
Predicted probabilities of antimicrobial resistance across the study period (2013–2021) considering poultry species (turkeys vs. chickens) and *Campylobacter* species (*Campylobacter jejuni* vs. *Campylobacter coli*). AZI, azithromycin; CIP, ciprofloxacin; CLI, clindamycin; ERY, erythromycin; GEN, gentamicin.

Turkeys had a higher probability of resistance to all examined antimicrobials compared to chickens (Table 2 and Figure 5).

Likewise, the odds of resistance to ciprofloxacin and nalidixic acid were higher in *C. jejuni* than in *C. coli,* but *C. jejuni* isolates showed significantly lower odds of being resistant to all the other antimicrobials when compared to *C. coli* isolates (Table 2 and Figure 5).

## Discussion

This study evaluated publicly available longitudinal surveillance data collected by the NARMS programme on AMR in *C. coli* and *C. jejuni* isolates of chickens and turkeys sampled at the slaughterhouse level across the USA over 9 years. We provided evidence-based data on the prevalence, patterns, and differences in AMR between *C. jejuni* and *C. coli* isolates and between turkey- and chicken-origin isolates.

A lower probability of resistance to all antimicrobials (except for tetracyclines), particularly macrolides (azithromycin and erythromycin) and quinolones (ciprofloxacin and nalidixic acid), was observed among *Campylobacter* isolates in the later years of the study (2019, 2020, and 2021). This finding agrees with two recent studies from the USA that showed a decrease in the probability of resistance to antimicrobials in *Campylobacter* isolated from chicken and turkey samples at retail [25] and slaughter [26]. Additional studies from the USA [27] and Canada [11] described a reduction of AMR in foodborne pathogens in chickens and turkeys. These are encouraging findings and might be related to the policy changes to AMU in poultry and livestock sectors, such as the veterinary oversight of the use of medically important antimicrobials in feed and water and the ban on using antimicrobials as growth promoters [28, 29].

The study results indicated that in both chickens and turkeys, *C. coli* isolates had higher AMR rates than *C. jejuni* isolates for most of the antimicrobials examined. This finding agrees with previous studies that described a higher proportion of MDR *C. coli* isolates from poultry [11, 15]. In contrast, it was described that *C. jejuni* isolates have a lower rate of AMR, but they better survive the food processing environment that aids them to enter the food chain and infect humans [30, 31].

A higher prevalence of resistance to all examined antimicrobials was observed in the *Campylobacter* spp. isolates derived from turkeys when compared to isolates derived from chickens. The longer production cycle of turkeys compared to chickens might increase the probability of contracting infections that necessitate antimicrobial treatment. This elevated exposure to antimicrobials might contribute to the higher AMR rates observed in turkey flocks. This finding agrees with a recent study from the USA, which described a higher level of AMR in *Salmonella* serovars of turkeys compared to isolates obtained from other food animals [27]. Future investigations are needed to identify factors and underlying reasons behind this issue.

The highest prevalence of resistance in both *C. coli* and *C. jejuni* isolates derived from chickens and turkeys was identified against tetracycline. This finding agrees with previous studies from North America and worldwide [9, 11, 14, 15, 32]. Tetracyclines are commonly used to treat and prevent bacterial poultry diseases [33], and the selection pressure of AMU is a major factor in the selection of tetracycline resistance. In 2018, tetracycline comprised 66% of the total antibiotics sold for administration to livestock and poultry in the USA [17, 31]. Also, genetic factors might contribute to the persistence and selection of tetracycline resistance as previous studies have indicated that mobile genetic elements carrying resistance genes can be transferred among various *Campylobacter* strains [31]. Earlier research [34] identified the plasmid-encoded gene *tet(O)* as the key determinant of tetracycline resistance in *Campylobacter.* A recent US study further reported that 13.2% of *Campylobacter* isolates from food animals carried the *tetO* gene [35]. Additionally, it has been documented that this gene can undergo horizontal transfer between *C. jejuni* and *C. coli* within the gastrointestinal tracts of food animals [36]. Apart from tetracycline use and genetic determinants, other factors contributing to the selection of tetracycline resistance should be further investigated.

Fluoroquinolones (e.g. ciprofloxacin) are the preferred empirical treatment choices for campylobacteriosis in humans [37], and the increase in fluoroquinolone resistance poses a public health risk. Similar to the findings of this study, high resistance to fluoroquinolones in *Campylobacter* was also previously reported among isolates in poultry, humans, and environment worldwide [9, 15]. The presence of fluoroquinolone-resistant *Campylobacter* is concerning, and to limit its emergence, the World Health Organization (WHO) included fluoroquinolone-resistant *Campylobacter* as a high-priority pathogen that requires increased research and development focus to advance new and effective antibiotic treatments [38]. In the USA, since 2005, fluoroquinolones have not been used in water to treat poultry bacterial infections [39], and the use of fluoroquinolones on turkey [40] and chicken [41] farms is limited, which points to the impact of non-AMU factors that influence the selection of fluoroquinolone resistance. Previous research studies have documented the presence of fluoroquinolone-resistant *Campylobacter* isolates in poultry in the absence of fluoroquinolone use [11, 16]. Moreover, a recent Australian study suggested that the infection source of fluoroquinolone-resistant *Campylobacter* in poultry might be attributable to humans, wild birds, or pests [16]. Cattle might also be a source for drug-resistant *Campylobacter* isolates in poultry as a recent Canadian study using molecular epidemiological methods showed genetic relatedness among cattle, poultry, and human *C. jejuni* isolates [42].

Biosecurity and farm management factors might also impact the prevalence of *Campylobacter* isolates in poultry flocks [43]. A recent Canadian investigation revealed that in chicken flocks the use of virginiamycin as a feed additive, using traps to control rodents, and the number of birds in a barn increased the prevalence of fluoroquinolone-resistant *C. jejuni* [44].

Macrolides (e.g. azithromycin and erythromycin) are the primary treatment choice for human campylobacteriosis [45]. Our results revealed a low prevalence of macrolide-resistant *Campylobacter*, apart from moderate resistance of *C. coli* isolates from turkeys, which is consistent with past observations of higher macrolide resistance in *C. coli* compared to *C. jejuni* in turkey flocks [11]. Macrolide resistance in both *C. jejuni* and *C. coli* is associated with point mutations in the 23S ribosomal RNA (rRNA) gene [46] and can also be conferred by the *erm(B)* gene [47]. Notably, in chickens, substitutions in the 23S rRNA gene (specifically A2075G or A2074C/G) have been linked to reduced colonization of flocks with *C. jejuni* [48]. Furthermore, it has been reported that the *erm(B)* gene is more frequently detected in *C. coli* compared to *C. jejuni* [49]. Further molecular-level investigations are needed to validate our findings.

*Campylobacter* isolates from both turkey and chicken caecal samples displayed low levels of resistance to gentamicin. Previous US investigations reported that *aph(2″)-Ig* and *aph(2″)-If* variants are the most predominant AMR genetic determinants associated with gentamicin resistance among *Campylobacter* [4, 46]. Historically, gentamicin was prescribed for the prevention of necrotic enteritis. However, the use of gentamicin in hatcheries (*in-ovo*) in the USA decreased between 2013 and 2019, with no reported usage after 2019 [50]. Our findings align with the mentioned intervention, showing a reduction in gentamicin resistance among *Campylobacter* isolates during the study period.

The cluster analysis of chicken isolates revealed similar AMR clusters in both *Campylobacter* species. However, *C. coli* exhibited a higher prevalence of tetracycline resistance, while *C. jejuni* showed greater resistance to ciprofloxacin and nalidixic acid. Given that *C. jejuni* is responsible for 80–90% of human campylobacteriosis cases [30, 31] and exhibits higher resistance to fluoroquinolones, it is crucial to intensify monitoring and investigations into the emergence of fluoroquinolone resistance, particularly in chickens, a main protein source for humans [51].

The cluster analysis of turkey isolates also indicated identical AMR and MDR clusters in *C. coli* and *C. jejuni.* The main distinction was that *C. coli* isolates displayed higher resistance to all tested antimicrobials, which could be explained by the inherent characteristics of this species, which is known to exhibit greater resistance to multiple antibiotics [30, 31].

Here, higher resistance to antimicrobials in *C. coli* isolates was observed in turkeys compared to chickens, suggesting that the former might play a larger role in the emergence of multidrug resistance in *Campylobacter* isolates. This finding is supported by a previous study, which suggested that antimicrobial-resistant *C. coli* might have the potential for better adaptation to the turkey farm environment and a higher tendency to colonize turkeys compared to *C. jejuni* [52].

The strongest pairwise correlation coefficients found here were observed between macrolides and quinolone classes in both *C. coli* and *C. jejuni* isolates from both poultry species. This may be explained by the cross-resistance within the same class of antimicrobials, facilitated by mobile genetic elements harbouring multiple resistance genes.

In addition to the NARMS programme in the USA, other countries have also integrated AMR surveillance systems to monitor indicator, foodborne, and pathogenic bacteria from poultry. These include the Danish Integrated Antimicrobial Resistance Monitoring and Research Programme (DANMAP), the European Antimicrobial Resistance Surveillance Network in Veterinary Medicine (EARS-Vet), and the Canadian Integrated Program for Antimicrobial Resistance Surveillance (CIPARS) [9, 13, 20, 53–56]. These programmes serve to identify emerging AMR trends and evaluate the effectiveness of antimicrobial stewardship strategies.

The present study is not without limitations. The absence of detailed information regarding the exposure history of turkeys and chickens sampled at the slaughter plants, including their on-farm AMU history and biosecurity and farm management factors, limited the ability to make direct links between the selection of AMR in *Campylobacter* isolates and the impact of various exposure factors.

In conclusion, we have shown a higher prevalence of resistance to most of the examined antimicrobials in *C. coli* isolates compared to *C. jejuni*, in both poultry species. Additionally, higher resistance rates were observed in *C. coli* and *C. jejuni* isolates obtained from turkeys compared to chickens. Over the study period, there was an overall decrease in the prevalence of resistance to the tested antimicrobials in *Campylobacter* isolates, particularly in the later years. Molecular epidemiological and on-farm studies are needed to acquire insights and promote understanding of the factors associated with the selection and persistence of antimicrobial-resistant *Campylobacter* isolates in the poultry production system.

## Supporting information

Sodagari et al. supplementary material Sodagari et al. supplementary material

## Data Availability

The data are publicly available, and we shared the link to the data set in the reference section.

## Supplementary material

For supplementary material accompanying this paper visit http://doi.org/10.1017/S0950268824000359. click here to view supplementary material
