# Supplementary material for: Monitoring antimicrobial resistance in Campylobacter isolates of chickens and turkeys at the slaughter establishment level across the United States, 2013–2021
Source: Epidemiol Infect. 2024 Feb 26;152:e41. doi: 10.1017/S0950268824000359 (PMC10945939; doi:10.1017/S0950268824000359)
Supplement: Sodagari et al. supplementary material [file S0950268824000359sup001.docx]

**Supplementary Table 1.** Minimum inhibitory concentration (MIC) breakpoints for *C. jejuni* and *C. coli* in seven antimicrobial agents*.*

| Antimicrobial class | Antimicrobial agent | MIC interpretive standard (μg/ml) ^a^ | | | |
| --- | --- | --- | --- | --- | --- |
|  |  | *C. coli* | | *C. jejuni* | |
|  |  | Susceptible | Resistant | Susceptible | Resistant |
| Aminoglycosides | Gentamicin | ≤ 2 | ≥ 4 | ≤ 2 | ≥ 4 |
| Lincosamides | Clindamycin | ≤ 1 | ≥ 2 | ≤ 0.5 | ≥ 1 |
| Macrolides | Azithromycin | ≤ 0.5 | ≥ 1 | ≤0.25 | ≥ 0.5 |
|  | Erythromycin | ≤ 8 | ≥ 16 | ≤ 4 | ≥ 8 |
| Quinolones | Ciprofloxacin | ≤ 0.5 | ≥ 1 | ≤ 0.5 | ≥ 1 |
|  | Nalidixic acid | ≤ 16 | ≥ 32 | ≤ 16 | ≥ 32 |
| Tetracyclines | Tetracycline | ≤ 2 | ≥ 4 | ≤ 1 | ≥ 4 |

^a^ Breakpoints were adopted from an epidemiological cut-off value
